# Angular Curvature of Spine Treated by the Paper-and-Glue Brace

**Published:** 1879-12

**Authors:** Bernard Bartow


### A CASE OF ANGULAR CURVATURE OF SPINE TREATED BY THE PAPER-AND-GLUE BRACE.

REPORTED BY BERNARD BARLOW, M. D.

Mention in the last number of your Journal, of the paper-and-glue spinal brace of Dr. Morgan Vance, has led me to think that the report of a case of angular curvature of spine in a child, two-and-a-half years old, in which this brace has been used, would be of interest to the profession, as exemplifying a few of many advantages claimed for this apparatus over that of plaster of Paris.

The patient has been attended jointly by Dr. Jno. Hauenstein and myself, and first came under the notice of the former gentleman the 9th of June last, when he observed an angular curvature of the spine of one-half inch projection posteriorly, involving the last dorsal and first lumbar vertebræ. The dorsal and lumbar curves of the spinal column were also to a large degree obliterated—the child's back presenting a flat appearance, from which the angle formed by the diseased vertebræ projected sharply. There was evidence of absorption of inter-vertebral substance.

The history of the case showed that it was one of an acute character and due to constitutional causes, not injury. The child had been backward in development and strength, never having walked, though able to stand with the support of a chair, etc. The mother noticed the curvature of spine three days previous to consulting Dr. H.; during three or four weeks previous to that time the child had had occasional diarrhœa, lost considerably in flesh and strength, and was irritable and restless in an unusual degree.

The obviously acute character of the disease, and the dependence upon it of the child's condition, suggested prompt measures for the prevention of deformity, if not of an unfavorable issue in the case. Being too young to be placed in a suspensory apparatus, the patient was suspended by the hands of an assistant placed in the axillæ. The plaster of Paris corset was then (June 12th) applied; care being taken to protect all prominent points, including curvature from pressure. The effect of this appliance was to relieve at once the irritability the child suffered through the unsupported spine, and although it was a heavy and hot appliance for one so young, and gave rise to a good deal of general discomfort, was otherwise well borne; with the addition of cod-liver oil and iron the patient improved perceptibly. The jacket was worn six weeks, and upon its removal a slight abrasion was found over the point of curvature, necessitating a delay of a week before another could be applied. During this time the child was kept upon her back and none of the advantage was lost that had just been obtained. No change had taken place in the degree of curvature. A second corset was applied and the child was then taken into the country, but it was necessary to bring her to the city, and remove the bandage at the end of three weeks, owing to an exploded percussion cap having, by some means, gotten between the corset and the patient's skin, causing a slight ulceration. On account of the difficulty of applying plaster of Paris in the case of one so young, the tenderness of the child's skin and the danger of continuous pressure upon prominent points, and the discomfort from wearing an apparatus in hot weather that did not admit of frequent removal and cleansing of the child's person, the necessity of some appliance admitting of these features was a desideratum with us.

The paper-and-glue spinal brace of Dr. Vance came to our notice at that time through the columns of the BUFFALO MEDICAL AND SURGICAL JOURNAL, and it was at once adopted and made, and when completed, found to be an elegant apparatus of its kind; combining the properties of firmness, lightness, and ventilation in a high degree; affording perfect support to the spine, and admitting of removal and re-adjustment with great ease. It might be mentioned here that the time necessary for its construction, as mentioned by Dr. Vance, is erroneous. Two persons of average expertness will consume at least four hours in the making of the cast and the subsequent application of the paper, glue and steel springs; but this is compensated for by the ease of application and longer time that the brace can be worn. Dr. V. states the actual working time to be one and a half hours.

This brace was applied to the patient August 15th, and has been worn two months; the mother has removed it every night, and has thus been able to bathe the child, greatly promoting rest and refreshment. It is applied before the child rises in the morning —in fact she refuses to move until it is put upon her, and cries for it when for any reason it is removed from her during the daytime. The good result of its use is seen in the complete arrest of the spinal curve, the ability of the child to walk by taking hold of another's hand, and increase in flesh, strength and good spirits.

The tender age of the patient, the acuteness of the disease, the greater readiness with which the apparatus was borne, and its being at least equally as efficient as the plaster of Paris bandage in controlling the disease, are strong recommendations for its use in cases which do not present as many difficulties as are met with in the treatment of this case.

